# River networks and funerary metal in the Bronze Age of the Carpathian Basin

**DOI:** 10.1371/journal.pone.0238526

**Published:** 2020-09-11

**Authors:** Paul R. Duffy

**Affiliations:** 1 The Italian Academy for Advanced Studies in America, Columbia University, New York, New York, United States of America; 2 The Archaeology Centre, University of Toronto, Toronto, Ontario, Canada; University at Buffalo - The State University of New York, UNITED STATES

## Abstract

Archaeologists use differences in metals from burial contexts to identify variation in social inequalities during the European Bronze Age. Many have argued that these social inequalities depended on access to, and control of, trade routes. In this paper, I model critical gateways in the Tisza river—a river system in the Carpathian Basin that might have enabled privileged access to metal in some areas but not others. I then evaluate the concentration of metal on different topological nodes of the river network in an attempt to understand what best explains the distribution of metals across this landscape. I do this by describing Bronze Age metal consumption and display in cemeteries from four micro-regions of the Tisza, and compare them with network ‘betweenness centrality’ values for locations along the river. I find support for the argument that favourably located river nodes had better access to metal in the earlier part of the Bronze Age.

## 1. Introduction

Explaining the origin, persistence, and variation in social inequality in time and space remains a central challenge for archaeologists [1, 2]. We know that social inequality must be institutionalized in middle-range societies before further demographic and political growth. Archaeologists often argue that different sources of power, such as religious institutions and economic infrastructure, are the key to maintaining and growing social inequality [3, 4]. Many believe it was warrior expeditions and control of metals and trade routes that enabled the rise of social inequality and the consolidation of power in Bronze Age Europe [5–12]. Yet in the Carpathian Basin, social inequality is variable; political economies seem to have emerged and co-occurred alongside more egalitarian societies [5, 13–16]. This paper investigates the possibility that trade along a key river offered people in some areas privileged access to metal, creating the potential for varying displays of inequality.

When investigating trade in the development of social complexity and inequality, ethnographers, archaeologists, and historians note the importance of location in trade networks [17–19, 20:100, 21]. Some archaeologists describe certain locations offering a ‘comparative advantage’ in trade relationships, and that these differences can be responsible for local social transformations and inter-regional inequalities [21–23]. The degree of advantage that a trade route offers is often measured by the degree to which human mobility is constrained by the physical properties of the landscape and the capabilities and technologies of individuals. These properties, or ‘affordances’, condition, and are conditioned by, culture change [24, 25]. One way of investigating these mobility constraints is to use network theory to model relevant dimensions of the geography [26–28]. Several network modeling studies [29–31] illustrate the utility of network approaches to evaluate social differences in the Carpathian Basin during the Bronze Age.

To situate my approach, I use Ray Rivers’ useful distinction between ‘theory models’ and ‘data models’ [32, 33]. For archaeologists, a theory model can take the properties of the environment, or landscape, and measure connectivity given several assumptions that are not informed by archaeological evidence. Data models do the opposite; they take the material culture itself and measure connective properties that are not informed by the environment or landscape. The same distinction is defined as ‘network modelling’ vs ‘network analysis’ by Östborn and Gerding [34]—I adopt the former here.

In this paper, I use network theory to investigate the relationship between variation in the accumulation and display of wealth and topological position on a river network in the prehistory of the Carpathian Basin. Here, long-lived tell settlements and trade seem to have crystalized along rivers [35–37]. I explore the idea that location on the river network impacts a community’s ability to concentrate and display bronze items in funerary contexts. I take the Tisza river and convert it into a network model (that is, a theory model) navigable by boat with some degree of portage over land. This differs in many respects from construction of least cost paths to model movement, which almost invariably model pedestrian travel on land [38, but see 50]. I then measure the betweenness centrality values of different locations on the network and compare them to the percentage of burials with bronze, the number of objects per capita, and the estimated bronze weight (wt) per capita in cemeteries during the latter part of the Early Bronze Age [c. 2200–1750 BC) and the Middle Bronze Age (c. 1750–1400 BC). I argue that such models provide a useful way to examine the relevance of travel routes on variation in access to metal wealth observed in the archaeological record. I find some evidence that accumulation and display of metal wealth in the Bronze Age of the Carpathian Basin corresponds to a topological position in the connectivity of a river, but that variables besides topology are also involved on more centrally located nodes.

## 2. Network and geographical models

### 2.1 Hydrology, trade, and canoe-based travel

The Great Hungarian Plain was an important crossroad in history and prehistory, connecting the Near East and the Balkans with the European continental interior. Large tell settlements emerged on the Great Hungarian Plain in the Late Neolithic and again in the Bronze Age, commensurate with long term occupation, populations in the hundreds or more, and participation in trade networks bringing exotic resources from distant parts of the Carpathian Basin and beyond [39–41]. Although these sites on the Plain are some of the largest known in European prehistory, the Plain has no naturally occurring resources such as workable flints or metal ore [42, 43].

Besides the utility of bronze, it is perhaps the rarity of the raw material, and the distance it had to travel, that made it one of the most identifiable forms of wealth in the Bronze Age. Gold, copper and copper alloys were used for fashioning body ornamentation and jewellery such as bracelets, beads, arm spirals, and headdresses [44]. Weapons, including axes, daggers, and lances, were common elements of Bronze Age lifestyle and death costumes [45]. Bronze was likely inalienable in some contexts and commodified in others, but in either case was symbolically charged [6, 46–48].

The most likely form of travel for people transporting unprocessed metal ore was by canoe along rivers [35]. Major settlements are almost invariably located on, or near, river channels, and settlements on these meanders may have been able to control the movement of ores and finished goods by monitoring traffic [8, 22]. Although it is possible that metal ore was transported on foot, large amounts of ore are needed to produce small bronze items, making foot-transport unlikely. Ox-driven carts are a possible solution, but the lack of good roads and the ubiquity of marshes would have made such passage difficult in many parts of the Great Hungarian Plain.

**Fig 1:**
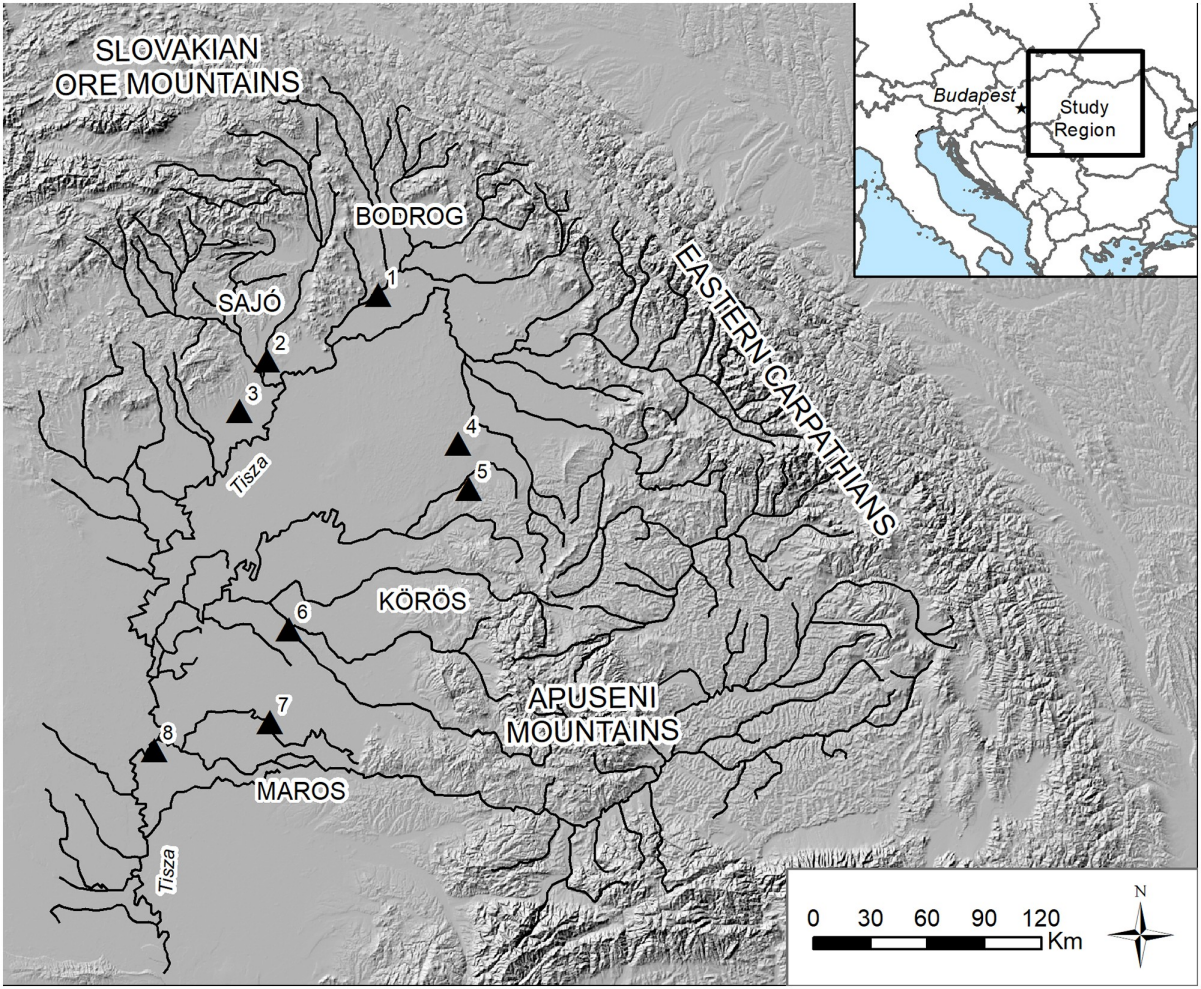
The study region with sites and catchments. Sites: 1, Streda nad Bodrogom; 2, Hernadkák; 3, Gelej; 4, Ciumesti; 5, Pir; 6, Békés 103; 7, Battonya-Vörös-Október; 8, Szőreg-C. Map by the author. Source of European base map layers: Esri. This work is licensed under the Esri Master License Agreement. Site locations, river vector, and tin dataset by the author.

The Tisza (*Tisa*, in Romanian) river catchment is the second largest river in Hungary after the Danube, draining the Slovakian Ore Mountains and Apuseni Mountains in Romania, funneling the water south into Serbia to ultimately join the Danube at Belgrade (Fig 1). The hydrological catchments of the Tisza river in the Carpathian Basin studied here, the Sajó (*Sajo* in Slovak), Bodrog, Körös (*Criș* in Romanian), and Maros (Mureș in Romanian), range in size. Each individual catchment had natural resources that travelled to other areas of the Carpathian Basin or outside of it, and each shows some similarities in material culture with neighboring areas suggesting shared traditions or interactions. Each has ores at higher altitudes, and each had larger fortified sites at lower elevations. The Sajó valley was the gateway to southern Poland and Scandinavian trade. The Upper Maros and Upper Tisza drained the interior of the Transylvanian Plateau, an enormous area. The Szamos (*Somes* in Romanian) and Bodrog had mountain passes to Ukraine. The Körös was mostly a large wetland in the heart of the Basin and drained the western Apuseni Mountains.

### 2.2 Using network theory for modeling river travel

Archaeologists and historians have often argued for the importance of geographically favorable locations in the development of ‘gateway communities,’ trade centers, and heightened inter-community interaction [17–19, 49, 50]. And when river-based trade is important, river confluence nodes provide varying degrees of importance, or betweenness centrality, for movement through the system. To understand the rise in prominence of Cahokia in the 11^th^ and 12^th^ centuries, Peregrine [49] converted the Mississippi river system into a network to evaluate the potential of different reaches to serve as mediators in the overall system. Of the forty-five confluences in the Mississippian river network, the confluence where Cahokia is found enjoyed the highest or second highest centrality according to several network measures.

The centrality of nodes in a network can be measured in different ways [51–54]. Betweenness centrality measures how well situated a node is in terms of the paths it lies on—that is, the higher the betweenness value, the more likely that the node must be crossed to get to another node on the network. In this sense, it is an appropriate metric for identifying good candidates for ‘gateway communities’ [sensu 17]. The betweenness values of nodes on the river system of the Carpathian Basin may be important because the river was anastomosing in very flat parts of the Plain in antiquity, and some parts of the network could have been very difficult to control because alternative routes were easily available (13:277]. This paper evaluates the topological position of different nodes in the Tisza river system, but uses a modified network that includes portage points in the calculations. In this sense, this paper comprises a social network analysis (as opposed to simple use of graph theory) because it models the ‘potential’ for social actors (communities) to achieve greater access to precious resources.

## 3. Methods

For this study, I digitized the Tisza hydrology, converted the hydrology to a matrix, calculated network values of nodes, and compared metal concentration values from cemeteries to network Freeman betweenness centrality values [54].

### 3.1 Source data and procedure

**Fig 2:**
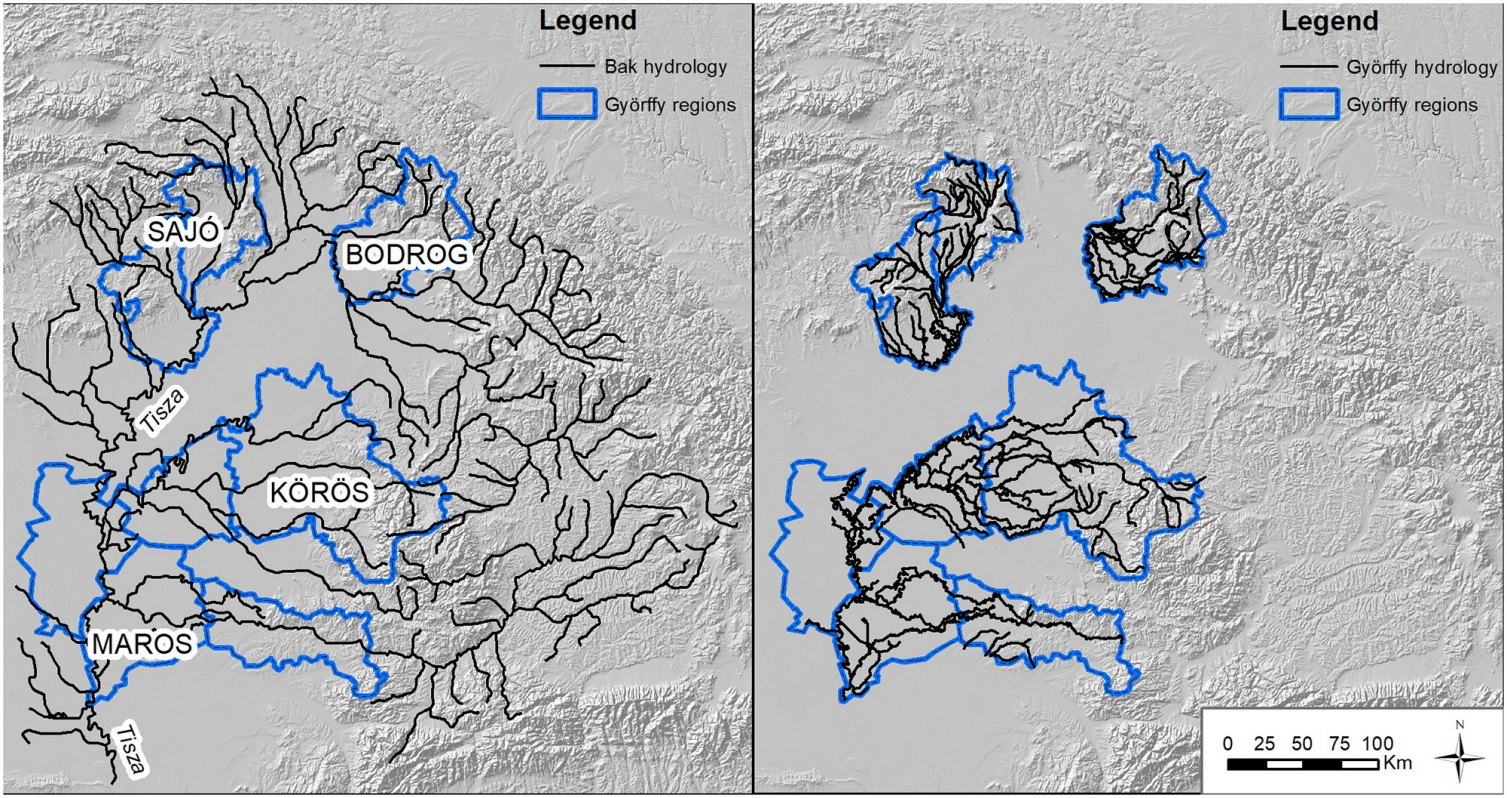
The Tisza river catchment and Great Hungarian Plain in the eastern Carpathian Basin using Bak’s data (left) and Györffy’s data (right). Map by the author. Site locations, river vector, and tin dataset by the author.

Digitizing the prehistoric hydrology of the Carpathian Basin requires piecing together datasets that predate the 1700s. During the 18^th^ and 19th centuries, engineers working for the Habsburg empire drained vast mashes on the Great Hungarian Plain, straightened the meandering channels, and built a complex artificial hydrological landscape composed of levees and dams [55, 56]. Archaeological site data indicate, however, that the river network of at least the Körös region had been stable since the Early Holocene until these recent, intensive modifications [57]. Any hydrological analysis over much of the Plain therefore requires working with reconstructed hydrology. In this analysis, I combined two cartographic sources of differing resolution developed by historical geographers Bak [58] and Györffy [59] to approximate pre-regulation hydrology for the Bronze Age (Fig 2). I then assembled these datasets into a single river.

Next, I restricted the analysis to reaches below 400 masl. I did this because inclusion of river features above 400 m would artificially lower the importance of nodes at lower elevations because the higher reaches were not points of settlement and trade. It was appropriate because Bronze Age people in the Carpathian Basin were predominantly wheat and barley farmers [60–62], and few sites are documented higher than this elevation.

Then I incorporated portage traverses into the model because land bridges or portages are logical alternatives to travel on water if the distance costs of river travel are high. Many of the river reaches and headwaters between different drainages are quite close to one another and would have only been a short portage across land. I identified the traversable portage routes as distances of 5 km, or less, as the crow flies. This is my educated guess of what a Bronze Age traveler may have considered to be a reasonable portage distance, approximately an hour of travel [63]. Finally, to keep the network analysis to computer processing times manageable with a desktop computer, I restricted nodes on the network to river confluences and every 10 km of a reach.

**Fig 3:**
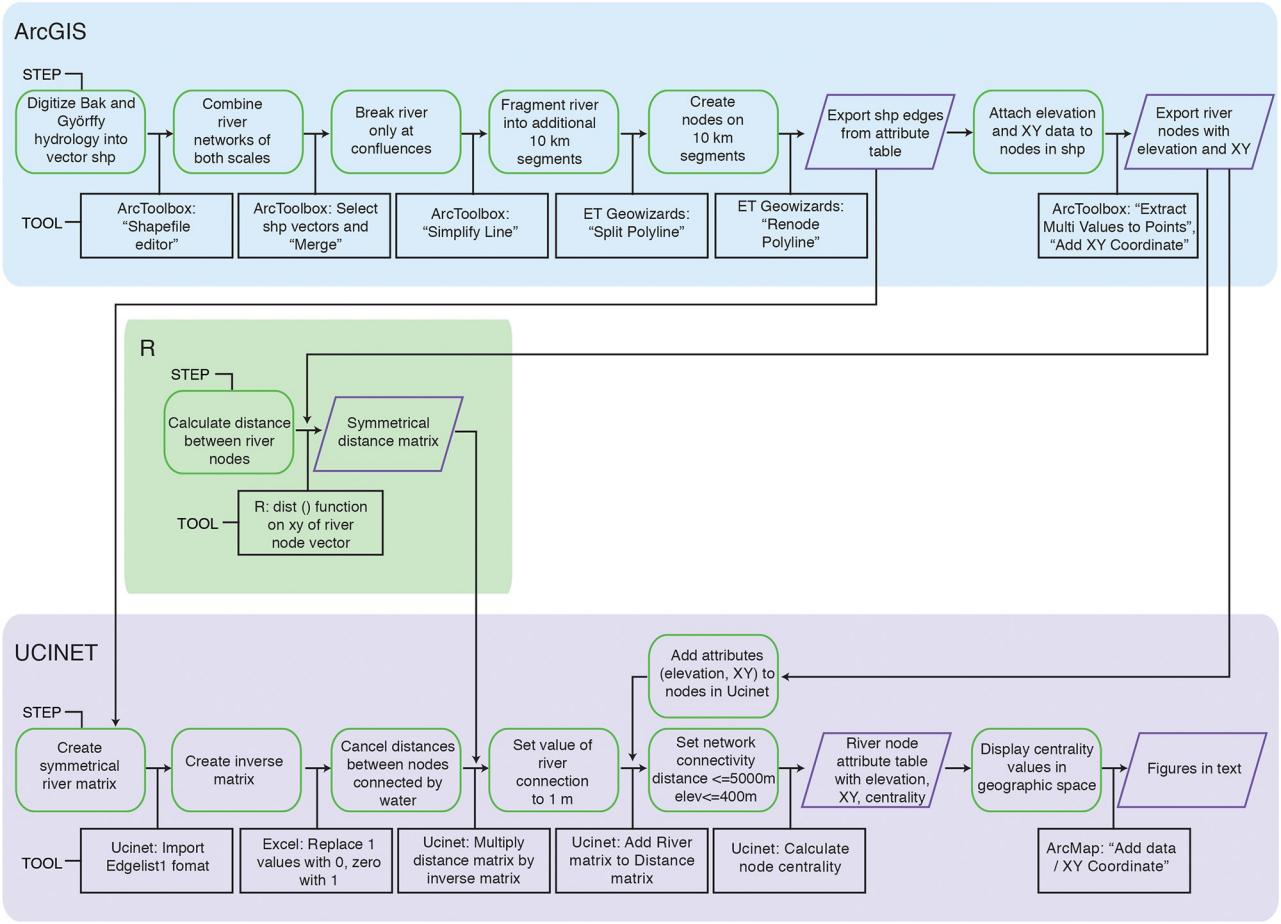
Workflow for generating betweenness centrality values of river nodes.

Archaeologists still lack user-friendly tools for modelling spatial networks [38]. Consequently, turning the Tisza river drainage into a network with node attributes and land bridge properties required working in ArcGIS, R, and Ucinet [but see 50 for another approach to a similar problem]. The process of data creation is outlined in Fig 3, illustrating the steps, the software tools used in conversion of datasets, and the outputs transferred between programs. I provide full methodological details in the supplementary document.

### 3.3 Archaeological datasets

Eight cemeteries were included in the study for comparison to river network values (Fig 1). As criteria for inclusion, I used published data for Early and Middle Bronze Age cemeteries with over 20 graves. The well-known cemetery of Mokrin [64] was excluded because the tributary on which it is located drains into the Tisza at the edge of the digitized network, causing network values to suffer disproportionate edge effects.

#### Battonya-Vörös Október MTSZ

The site is located north of the town of Battonya in Hungary, on the Száráz Ér, a small tributary to the Maros river. It was excavated between 1964 and 1966 and also between 1973 and 1979 [65, 66]. It has approximately 130 graves (135, including multiple burials), with both inhumations and cremations. Radiocarbon dating places the cemetery between 1950 and 1800 BC [67].

#### Békés 103

The cemetery is on the northern edge of the town of Békés in Hungary, near the confluence of the Fekete and Fehér Köros Rivers (*Crișul Negru* and *Crișul Alb* in Romanian). It was excavated between 2011 and 2019, and has 68 graves, mostly cremations, which have been published and extensively radiocarbon dated [68–71]. The site was in use between 2460 and 1010 BC, but the most intensive period (Phase 4) was between 1600 and 1280 BC. A few Early and Late Bronze Age burials are found at the site, but here I include only the burials with Middle Bronze Age style ceramics (Phase 4), or dated to the Middle Bronze Age (HB 14 and 21), for analysis.

#### Ciumești

The site is near the town of Ciumești in northwestern Romania is almost equidistant between the Kraszna (*Crasna* in Romanian) and Ier rivers (the Crasna drains into the Tisza, while the Ier drains into the Körös) [72, 73]. Twenty-six graves, all cremations, were excavated between 1962 and 1965. Although the site has not been radiocarbon dated, the ceramics date to the Otomani I phase, or latter part of the Early Bronze Age (c. 2100–1700 BC).

#### Gelej

The cemetery is in northeastern Hungary on the Csincsa stream—a small tributary to the Upper Tisza river, near the village of Gelej. It was excavated first in 1941, then again in 1962, and 1966–1968 [74]. There are 171 graves, all inhumations, attributed to the Middle Bronze Age Füzesabony culture (c. 1700–1400 BC). A large number of Late Bronze Age burials were also excavated from this site, but are not included here.

#### Hernádkak

The cemetery is 15 km from the city of Miskolc, and 2 km from the Hernád river [75]. It was excavated in 1934 and 1935, and approximately 132 graves recovered, mostly inhumations. The site has not been radiocarbon dated, but the ceramics date to approximately 2200–2000 BC. Copper or bronzes noted but not relocated by Schalk [72] in the catalogue of finds are included in the study.

#### Pir

The cemetery is near the village of Pir in northwestern Romania, 3 km from the Ier River. The site was excavated from 1953 to 1954 and again in 1977 [76, 77]. There were 31 graves, and all but one cremation, are inhumations. The site has not been radiocarbon dated, but most of the ceramics fall within the Middle Bronze Age (Otomani II), c.1700-1400 BC.

#### Streda nad Bodrogom

The cemetery is in southern Slovakia near the Bodrog river, and was excavated in 1955 [78]. There were 67 graves, with inhumations and cremations in similar proportions. The ceramics date the cemetery to c. 1700–1400 BC.

#### Szőreg-C

The cemetery is located in the village of Szőreg in Hungary at the Tisza-Maros confluence. Approximately 230 inhumation graves were excavated between 1928 and 1930 [79, 80]. P. Fischl [81, 82] revisited the field notes from the excavation and corrected some inconsistencies, published the cemetery map and refined the chronology [83]. Several graves were radiocarbon dated and fall between 2100 and 1600 BC [67].

Body treatment may have an impact on the presence of metal in mortuary contexts, as cremation was a common practice in some parts of the Great Hungarian Plain. Although metal objects are sometimes placed inside burial urns, metal objects could have been placed on the pyre and not been recovered for burial with the dead. For this reason, I initially present the results grouped together, but then present the data separated according to cremation and inhumation. Rare body treatments such as scattered cremations and symbolic graves are excluded. Where multiple individuals could be identified with corresponding grave goods, I considered these independently.

Some authors have noted that the percentage of burials with bronze on the eastern Great Hungarian Plain seems to vary over time [8, 80:344, 84:62]. For this reason, I broadly classify the burials in the cemetery sample into temporal phases and consider this variable as possibly affecting metal concentration. At Szőreg-C, which was in use during multiple Bronze Age phases, burials were excluded if they could not be assigned to a particular phase [see 81, 82]. My analysis excludes graves in all cemeteries that are recorded as ‘disturbed’ or ‘destroyed’, and only those burials from Békés 103 with preservation recorded as ‘Good’ or ‘Very Good’ [68]. See S1 Table in S1 File for the full list of burials included.

### 3.4 Metal numbers and metal weights

**Fig 4:**
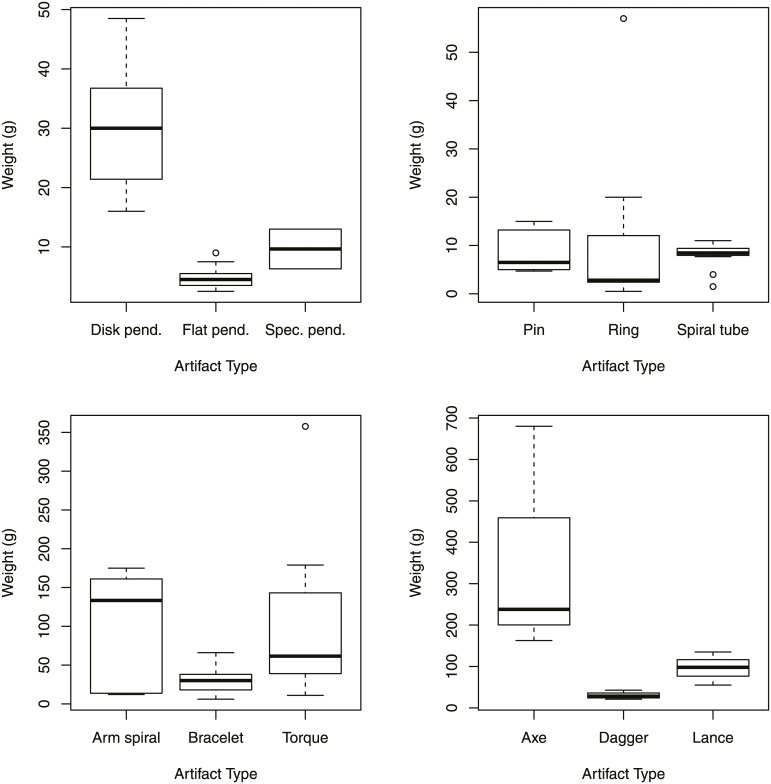
Selected artifact classes by weight. For n see Table 1.

I consider here metal objects, such as multi-bead necklaces as unitary artifacts and not composite artifacts due to the ambiguity of objects often present in the mortuary record. I assume that object types and compositions were symbolically important and would have been meaningful for different social roles and cultural circumstances. As a commodity however, copper and bronze can be melted down, and the absolute amount of metal found in graves and cemeteries has arguable significance in terms of the ability to display metal wealth. Here I devise several metrics to categorize and compare the concentration of metal wealth as a way to gauge access to, rather than meaning of, the metal objects. I refer to the objects as ‘bronze’ for convenience, as many of the metals included in the analysis may be unalloyed coppers [85]. I also estimate the weight of objects in the cemeteries based on object weights from other sites, as the published artifact descriptions for the sites used in the study do not contain weights. Many of the bronze object types found at sites, such as Nagycenk-Lapos-rét and others, however, are also found at cemeteries in this study [86: 60]. The published weights for artifacts from several sites are summarized in Table 1 (raw data in S2 Table in S1 File), and the distributions of object weights are shown in Fig 4. Several classes of artifact only have a single object weight (or no object weight) and many of the artifact classes have a broad range of weight values. I provide a ‘Study Estimate’ nonetheless, as I considered this better than using the number of unitary objects alone in order to gauge differing access to the metal as a commodity.

**Table 1:** Standardized weight estimations based on S2 Table in S1 File (artifact weights).

Artifact Type	n	Average Weight (g)	Std Dev.	Study Estimate	n (this study)
Button	1	1.0		1	5
Bead				1	18
Flat pendant	14	4.8	1.8	5	15
Unidentified				5	1
Spiral tube bead	12	8.0	2.6	8	125
Pin	6	8.5	4.5	8	88
Titulet				8	321
Spectacle pendant	2	9.7	4.7	10	3
Earing				10	2
Ring	7	12.7	20.6	13	29
Awl				15	6
Chisel				15	1
Spiral	1	21.0		21	7
Bracelet	20	28.9	14.2	29	20
Flat disk pendant	11	29.8	10.2	30	321
Dagger	4	30.0	9.1	30	10
Flattened sheet	2	43.5	47.4	44	1
Lance	3	96.0	40.0	96	3
Torque	16	97.8	88.9	98	2
Arm spiral	6	104.7	74.9	105	3
Axe	3	360.2	279.5	360	4
Grand Total	109	46.0	83.8		

### 3.5 Comparison of cemetery metrics to river metrics

If river nodes near cemeteries were important for defining the use of metal in communities burying their dead, access to nodes of high network centrality would have to be within a reasonable distance. To identify these nodes, I created a 5 km buffer around each site and used it to clip the hydrological dataset, selecting all river nodes within the area around a cemetery. From these nodes, the highest value was selected. It is often unclear where the settlements of the cemetery’s dead were placed, but it is assumed to be nearby [see 68, 87, 88].

## 4. Results

### 4.1 Network representation

**Fig 5:**
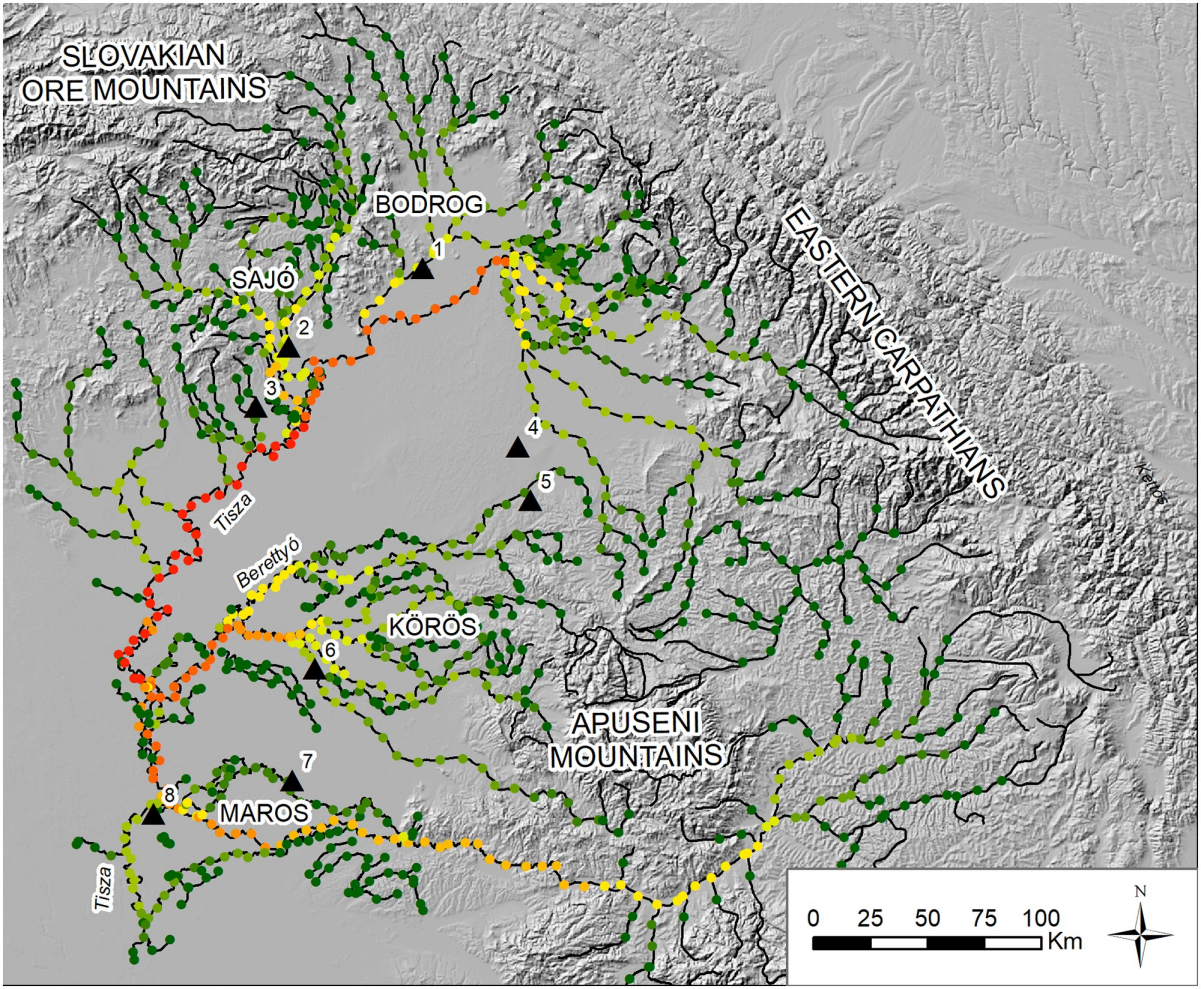
Combined Bak and Györffy hydrology, with 10 km segment nodes. Colour (red = high and green = low) indicates betweenness centrality scores for nodes on a network with 5 km land bridges and no nodes above 400 masl. Sites: 1, Streda nad Bodrogom; 2, Hernadkák; 3, Gelej; 4, Ciumesti; 5, Pir; 6, Békés 103; 7, Battonya-Vörös-Október; 8, Szőreg-C. Map by the author. Site locations, river vector, and tin dataset by the author.

Networks created without any land bridges have their highest betweenness centrality values along the main artery of the Tisza. Once 5 km land bridges are introduced to the network, the pictures shift quite dramatically because the Körös region then includes the highest betweenness values—the Sebes Körös (*Crișul Repede* in Romanian) specifically, because it joins up with the Szamos catchment at the northern base of the Apuseni Mountains. Restricting the model to nodes under 400 masl prevents connectivity in the headwaters of the Tisza, however, reduces the Körös basin connectivity—except for the Berettyó, which sees slightly higher connectivity than the rest. The geographic representation of the network in Fig 5 is modified according to these final parameters. It includes the combination of Bak and Györffy’s maps, with edges between nodes every 10 km. It also has the potential for travel across land bridges separating nodes by less than 5 km and includes only nodes under 400 masl (see S3 Table in S1 File for a list of the nodes with XY coordinates and betweenness centrality values). Experiments varying the length of the link between nodes, the number of nodes, and the straightness of the river channels suggest these variables are not particularly important for the resulting network values.

### 4.2 Comparison of betweenness centrality to metal values in cemeteries

Due to the high degree of disturbance in the cemeteries, only 65% of the graves could be included in the study. The bronze values for the graves in the sample are presented in Table 2. Bronze metrics in Tables 3 and 4, where each body treatment is treated separately, seem to illustrate that body treatment at death has a strong effect on the resulting values. When the sample is restricted to cemeteries with more than 20 burials, the average percentage of inhumation burials with bronze is 0.22, the per capita bronze is 1.19 objects, and the per capita bronze wt is 12.65 g (n = 6). By contrast, the average percentage of cremation burials with bronze is 0.07, the per capita bronze is 0.15 objects, and the per capita bronze wt is 1.79 g (n = 3).

**Table 2:** Summary of cemetery burials included in the study.

Site	Total burials	Bronze Age Phase	Intact burials in sample	Bronze objects	Bronze weight (g)	Intact burials with bronze (percent)	Bronze per capita	Bronze wt per capita
Battonya Vörös Október	135	Early	71	15	593	0.11	0.21	8.35
Békés 103	63	Middle	22	3	45	0.14	0.14	2.05
Ciumești	26	Early	14	0	0	0.00	0.00	0.00
Gelej	170	Middle	107	29	206	0.13	0.27	1.93
Hernádkak	132	Early	131	195	2758	0.38	1.49	21.05
Pir	31	Middle	25	3	39	0.12	0.12	1.56
Streda nad Bodrogom	67	Middle	46	17	143	0.13	0.37	3.11
Szőreg-C (Early)	231	Early	108	120	1296	0.31	1.11	12.00
Szőreg-C (Late)		Middle	31	115	962	0.23	3.71	31.03
**Total**	**855**		**555**	**497**	**6415**			

**Table 3:** Metal concentration in inhumation burials only.

Site	Intact inhumation	Inhumations with bronze	Inhumation bronze objects	Inhumation bronze wt	Percentage of inhumations with bronze	Inhumation bronze per capita	Inhumation bronze wt per capita
Battonya Vörös Október	48	7	13	563	0.15	0.27	8.35
Békés 103	2	1	1	8	0.50	0.50	2.05
Ciumești	0	0	0	0	0.00	0.00	0.00
Gelej	107	14	29	206	0.13	0.27	1.93
Hernádkak	129	50	195	2758	0.39	1.51	21.05
Pir	24	3	3	39	0.13	0.13	1.56
Streda nad Bodrogom	17	4	9	79	0.24	0.53	3.11
Szőreg-C (Early)	107	33	120	1296	0.31	1.12	12.00
Szőreg-C (Late)	30	7	115	962	0.23	3.83	31.03
**Total**	**464**	**119**	**485**	**5911**	**0.22**	**1.19**	**12.65**

**Table 4:** Metal concentration in cremation burials only.

Site	Intact cremation	Cremations with bronze	Cremation bronze objects	Cremation bronze wt	Percentage of cremations with bronze	Cremation bronze per capita	Cremation bronze wt per capita
Battonya Vörös Október	23	1	2	30	0.04	0.09	1.30
Békés 103	20	2	2	37	0.10	0.10	1.85
Ciumești	14	0	0	0	0.00	0.00	0.00
Gelej	0	0	0	0	0.00	0.00	0.00
Hernádkak	2	0	0	0	0.00	0.00	0.00
Pir	1	0	0	0	0.00	0.00	0.00
Streda nad Bodrogom	29	2	8	64	0.07	0.28	2.21
Szőreg-C (Early)	1	0	0	0	0.00	0.00	0.00
Szőreg-C (Late)	1	0	0	0	0.00	0.00	0.00
**Total**	**91**	**5**	**12**	**131**	**0.07**	**0.15**	**1.79**

Among the inhumations dataset, Hernádkak has the highest percentage of burials with bronze (0.39), and the second highest bronze object and bronze wt per capita. Szőreg-C has a lower percentage of burials with bronze (0.23–0.31), but still has higher bronze object and bronze wt per capita counts than Hernádkak (but only in the Middle Bronze Age). Szőreg-C triples its inhumation bronze and bronze wt per capita from the Early to the Middle Bronze Age.

**Fig 6:**
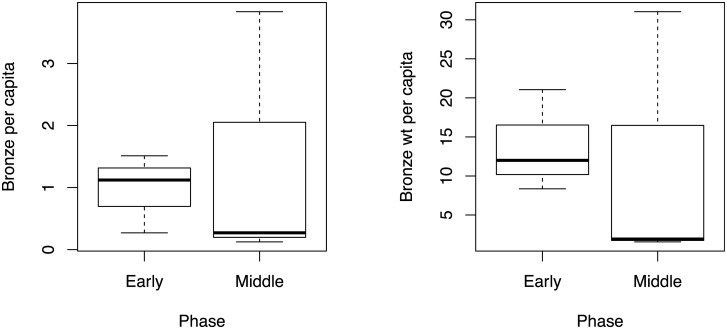
Bronze per capita and copper weight per capita in the early and Middle Bronze Age (inhumations only; Békés 103, Ciumești, and Streda nad Bodrogom are excluded).

**Fig 7:**
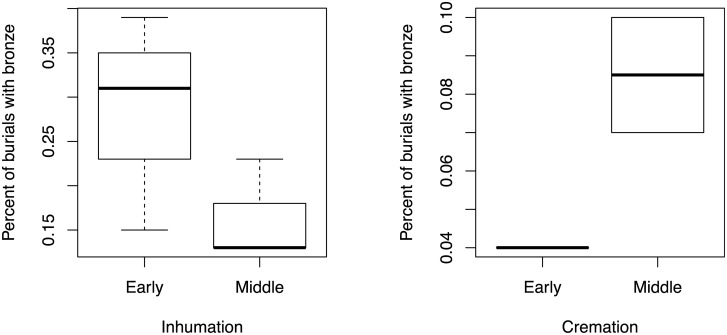
Percentage of burials with bronze in the early and Middle Bronze Age.

Metal objects and wt per capita are displayed for inhumation only in Fig 6 (including only cemeteries with 20 or more intact burials). In Fig 7, I present the percent of burials with bronze according to burial treatment and phase. Restricting the sample to cemeteries with more than 20 intact burials leaves the cremation plot with only one Early phase and two Middle phase cemeteries. Even for inhumations, the sample size for the Early phase is 3 cemeteries, and the Middle phase is 4 cemeteries.

**Fig 8:**
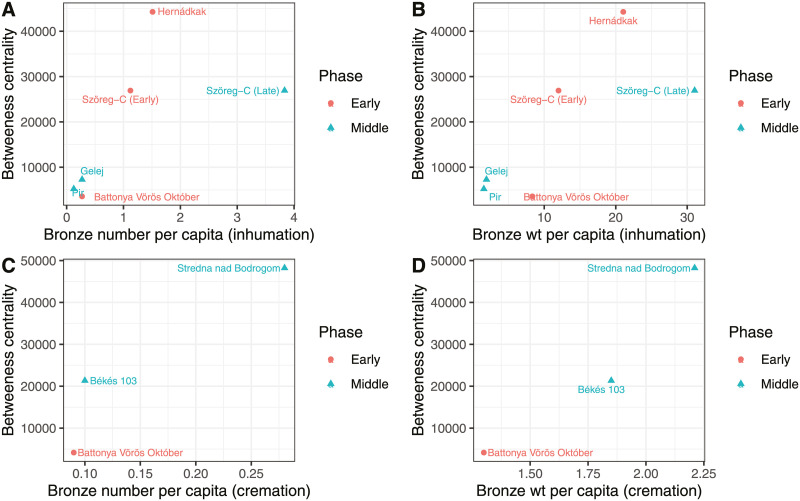
Bronze numbers and bronze weight per capita according to Middle Bronze Age phase.

**Fig 9:**
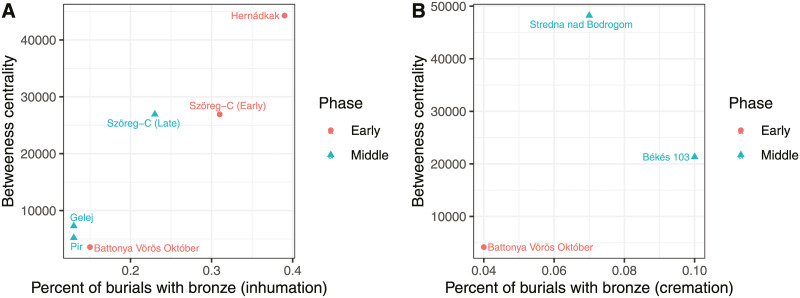
Percentage of burials with metal according to Middle Bronze Age.

The relationship between bronze number and wt per capita, percent of burials with bronze, and betweenness centrality is displayed by body treatment in Figs 8 and 9. Again, the sample size is small, but the correlations are instructive.

The average percentage of inhumation burials with bronze drops from 0.28 in the Early Bronze Age to 0.16 in the Middle Bronze Age. The mean bronze per capita and the mean per capita wt also drop from the Early to Middle Bronze Age, though the ranges become more expanded. Szőreg-C, however, is an outlier as there is an increase in bronze per capita and per capita wt, but a drop in the number of burials with bronze. Here, there is an overall increase in bronze objects per person (in the whole cemetery) deposited between the Early and Middle Bronze Age. Because the percentage of people buried with bronze drops in the Middle Bronze Age, however, it is actually the same amount of bronze going into fewer burials, and therefore overall, can be seen as an increase in the display of social inequality.

The number of cremation cemeteries in the sample is too low to evaluate against betweenness centrality, but there is a moderate to strong positive correlation between the various metal concentration metrics and betweenness centrality for cemeteries with over 20 inhumations when both Early and Middle phases are combined (Table 5). For bronze object per capita, and bronze wt per capita, there are moderate positive correlations with p-values above 0.05. The correlation between betweenness centrality and the percent of inhumations with bronze, however, is very strong at 0.97, p = 0.002.

**Table 5:** Pearson’s correlation coefficient values for betweenness centrality of river nodes plotted against two metrics of metal concentration in the cemeteries.

Bronze metric	Betweenness centrality	p-value
Percent of inhumations with bronze	0.97	0.002
Per capita bronze for inhumations	0.59	0.214
Per capita bronze wt for inhumations	0.74	0.091

## 5. Discussion

This paper models critical gateways in the Tisza river drainage and evaluates the concentration of metal on different topological nodes of the river network in an attempt to understand what parameters best explain the distribution of metals across this landscape. I found that proximity to important nodes in the river system is a good predictor of metal abundance in cemeteries. The capacity of people in a region to disproportionately benefit in exchanges because of their access to resources has been described as a ‘comparative advantage’ [21–23]. My results suggest that topological location on river networks should be included in the set of parameters that archaeologists use to identify and assess comparative advantages in the Bronze Age on the Great Hungarian Plain. To illustrate this, I draw the reader’s attention to Gelej and Hernádkak, which demonstrate vastly different metal profiles and yet both sit in the foothills of the Slovakian Ore Mountains, and both equidistant from the Tisza (Fig 5). My data suggest that Hernádkak on the Sajó enjoyed a much larger river network reaching into the mountains (and therefore received a high betweenness centrality score) than Gelej. This could have been responsible for the substantially greater amount of metal displayed in Hernádkak mortuary contexts. In contrast, the Csince stream, where Gelej is found, despite leading upward into the ore producing mountains, has many fewer reaches connecting the region to the wider river network of the Tisza (it therefore received a lower betweenness centrality score), and this could explain why it has less metal.

Aside from the observation connecting river topology to metal, these results suggest more detailed quantitative descriptions of metal should be involved in understanding the changing relationship between burial displays and social inequality. Archaeologists working on the Great Hungarian Plain have noted that the percentage of burials with metal-wealth drops from the Early to Middle Bronze Age. They suggest that the disappearance of metal from burials could be down to a shift to displaying wealth inequalities during life [80, 84]. This study found, however, that although the percentage of burials with metals does drop over time, the per capita metal inclusion in cemeteries is quite variable across the Great Hungarian Plain. For instance, the percentage of those inhumations buried with bronze at Szőreg-C dropped in the Middle Bronze Age from 0.31 to 0.24 but the per capita bronze and bronze wt nearly tripled. That is a stunning increase in bronze display, and quite at odds with the general trend of decreasing metal values elsewhere. Although social inequality may have shifted to being displayed during peoples lives, the variation in metal deposition between late Szöreg/C and other sites in the sample merits further investigation. The use of estimated bronze weight, a novel practice introduced here, also provides a balance to bronze object number and allows a greater confidence in using metal counts as a proxy for social inequality.

Does the pattern of river topology and metal hold across time? As a theory model, network metrics are chronology-free and betweenness centrality may work well as a predictor of metal concentration in some periods but not in others. I did not have enough data points to evaluate whether the importance of node centrality strengthened or weakened across time. However, there are reasons to believe that with adequate data, the relationship found in Bronze Age might apply to other periods. For instance, it may work well for the Late Neolithic and Early Copper Age, as spectacular and unusual sites of culture confluence coincide with Tisza betweenness centrality values. The site of Polgar-Csőszhalom (well-known for combining elements of the Lengyel, Herpály, and Cucuteni-Tripolye archaeological cultures), and the Early Copper Age cemetery of Tiszapolgár-Basatanya, are located on nodes of the Sajó near Tiszafüred. In this study, both are identified as within the top 2% highest betweenness centrality. The boundary between the Late Neolithic Tisza and the Herpály culture areas, where a lot of interaction would have happened, is also the Sebes Körös [89, 90], the point of highest connectivity in the Körös River network.

Other researchers have made clear however, that by the Late Bronze Age (c. 1300 BC), the importance of the river network for communication, travel, and trade may have been diminished [35, 91]. Late Bronze Age mega-sites such as Csanádpalota-Földvár and Cornești Iarcuri are off the Maros—a major waterway for travel and trade earlier in the Bronze Age [92–94]. In the Late Bronze Age and Early Iron Age, people leave the rivers of the Körös region for higher, drier ground, ending a settlement tradition present since the first farmers moved into the Basin in the seventh millennium BC [91]. There is no reason to believe that the hydrological model provided here would serve any predictive power in these new cultural contexts.

Because river-based travel is not a feature necessarily restricted to certain periods or parts of the world, there is also reason to believe the network method outlined here could be effectively applied to river drainages in other regions. River networks can structure trade whenever transport or travel by boat are important, and many landscapes across the world are most easily traversed on water, even though people are living on land nearby. The Danube, the Niger, and the Amazon would all make appropriate case studies, but as this study shows, smaller water catchments are equally appropriate given the key assumption of the model, that of predominant river travel was met during the period under study.

## Supporting information

S1 File Duffy 2020 river networks and funerary metal supplementary tables. (XLSX)

S2 File Duffy 2020 river networks and funerary metal supplementary document. (DOCX)
