# Supplementary material for: River networks and funerary metal in the Bronze Age of the Carpathian Basin
Source: PLoS One. 2020 Sep 11;15(9):e0238526. doi: 10.1371/journal.pone.0238526 (PMC7485878; doi:10.1371/journal.pone.0238526)
Supplement: S2 File — (DOCX) [file pone.0238526.s002.docx]

**Supplemental**

*S.1 Historical cartographic sources*

For the coarser resolution, I use Bak’s Carpathian Basin map based on pre-20^th^ century historical sources to create a hydrological network (1). Each reach was digitized and given its own object ID (n=264). For the finer resolution, I use the smaller scale medieval period hydrological maps by Györffy, who exhaustively detailed the locations of settlements, toponyms, and river channels from 14^th^ century historical records (2). These maps are detailed but restricted to certain areas. They nonetheless cover the regions of interest in the Tisza drainage system fairly well, and most of the towns present on the 14^th^ century maps are still present today. These maps were scanned and georeferenced in ArcGIS for digitization, using control points identifiable on modern 1:10 000 topographic maps. Comparison with other data sources (see 3) indicates the accuracy of river channel position of the historical geographical datasets is generally within 1 km.

*River segment length and land bridges in the network*

Modelling likely portage transfers requires both a point at which one pulls the canoe out of the water, and a point at which the canoe goes back into the water in another river. In network terms, this requires nodes not only at the river confluences shown in Fig. 3, but also on the river segment itself, potentially canoe livery points. The river network shown in Fig. 2 must therefore be segmented at much shorter intervals. If segment distance were 1 km, for example, minimally every 1 km a canoe-based traveler would have the opportunity to pull the canoe out of the water and portage to another river.

This threshold portage distance must also be defined. If portage distance were 5 km, for example, any time the distance between two nodes fell to 5 km or less, a link would be created between them. These portage points end up being potentially much more important in the network than river confluences because they similarly share the ability to act as central nodes in the overall network.

A technical difficulty arises however, when the portage distance is greater than the river segment length. This is because naturally, a land bridge threshold of 5 km connects the river to itself much more often when the river segment is the same length or shorter. Although this pattern is more or less consistent across the entire network, it creates a lot of noise in network values. In a general sense, the greater the number of nodes and connections in the system, the more complex the pattern and more difficult to interpret. A compromise between the segment length (geographical precision of potential livery points) and the distance in portage (realistic transport values) is therefore required. A balance must be achieved where the quantity of information is enough to provide patterns and hypotheses, but not so great to produce only spurious results. In an exploratory investigation such as this I proceeded through trial and error as much as by first principles. As geographical inputs of network models are modified, a general sense of the impact on overall network values on the models can be compared to one another and understood at a systemic level.

*S.2 Converting the river polyline to a matrix*

For the river network, nodes are defined when two or more reaches come together, or if two or more rivers drain into a marsh, and come out as a single reach downstream. The hydrology at both scales was digitized into polyline shapefiles and then combined by attaching vector lines of both scales, using the lower resolution data where the higher resolution data was not available^[[1]](#footnote-1)^. I then simplified the river geometry into single polylines breaking only at river confluences, eliminating any trace of the vectorization process. These confluences would become one kind of node in the river network.

The assumption of the model is that land bridges and portage would have been used in places where crossing distances were short. This therefore requires “leaving” the river to cross land to join another river. To represent this potential, additional nodes were added to the river network at 10 km intervals. The choice of 10 km as an interval was a compromise between allowing actors to leave one river channel to join another at any point, and the computational reality that having very short intervals radically increases the size of the matrix and the computational time in network software. Adding nodes to the river polylines was accomplished in two steps using *ET Geowizards*, first by splitting the polyline into 10 km segments and then by creating a point shapefile comprised of the beginning and ending vertices of the polyline segments using ‘Renode Polyline’^[[2]](#footnote-2)^. I then obtained the geographic coordinates (easting and northing in meters, from a UTM projection) and elevation (masl, from a SRTM raster) of each point in the shapefile, which serve as attributes for the nodes in network software (4).

In the process of creating a node shapefile, the ‘Renode Polyline’ Wizard application in *ET Geowizards,* also adds two columns to the original polyline shapefile, the ‘ET_FNode’ and ‘ET_TNode’ columns. These values refer to the nodes in the created point shapefile, essentially a list of edges for an adjacency matrix (that is, two columns specifying the connected nodes). This was exported as a text file for manipulation in UCINET.

The next step is the creation of a distance matrix for a vector of XY (easting and northing) pairs. This was accomplished by taking the locational coordinates of each river node from the ArcGIS output and producing a matrix using the stock dist() function in R. The output is a matrix in meters between each node.

The remaining operations were carried out mostly in Ucinet and NetDraw, simple network software available for matrix analysis and display (5). The edges of the river network were imported to Ucinet and then exported as a 2D array for manipulation in Microsoft Excel. In this matrix, river nodes are either connected (1) or they are not (0). To achieve a matrix with connections identifiable as river or land, however, a multiplier matrix was created in Excel. In this ‘Inverse’ matrix, produced using the ‘Find and Replace’ function in Excel, river connection cells are zero and all other cells are one. Using the ‘Command line / matrix algebra’ tool in Ucinet, the ‘Inverse’ matrix is multiplied by the distance matrix to cancel out the distance values where there are river connections. The original river matrix is then added to the distance matrix using the ‘Command line / matrix algebra’ tool. In this network, all nodes are connected to each other by ‘1’ or higher values representing the Euclidean distance between nodes in meters.

By adding the attribute data (XY and elevation) to the nodes in the network and displaying in NetDraw, the elevation of the nodes to participate in the network analysis is set to equal 400 m or less, and the relational value between nodes is set to equal or less than 5000 m. The resulting connections therefore are restricted to nodes that are at or below 400 m above sea level, directly connected by water (relation = 1), or accessible by a land bridge 5km or less (relation <= 5000m).

In Netdraw, the betweenness centrality is calculated using the ‘Centrality measures’ tool in the Analysis panel. The data can be exported from the Node Attribute Editor and then reimported to ArcGIS for visualization and comparison to archaeological datasets.

**References**

1. Bak Bl. Magyarország történeti topográfiája : a honfoglalástól 1950-ig. Budapest: Historia- MTA Történettudomány Intézete; 1997.

2. Györffy G. Az Árpád-kori Magyarország történeti földrajza. Budapest: Akadémiai Kiadó; 1966.

3. Gyucha A, Duffy PR, Frolking T. The Körös Basin from the Neolithic to the Hapsburgs: Linking Settlement Distributions with Pre-Regulation Hydrology Through Multiple Data Set Overlay. Geoarchaeology. 2011;26(3):293-419.

4. Farr TG, Rosen PA, Caro E, Crippen R, Duren R, Hensley S, et al. The Shuttle Radar Topography Mission. Reviews of Geophysics. 2007;45(2).

5. Borgatti SP, Everett MG, Freeman LC. Ucinet for Windows: Software for Social Network Analysis. Harvard, MA.: Analytic Technologies; 2002.

1. For example, because the Maros river is so wide on the Plain, the resolution was not very different between the two. For the Körös region, where the resolution is quite different because of the large area of anastomosing rivers, the picture is quite different. [↑](#footnote-ref-1)
2. This procedure could be accomplished with tools native to ArcToolbox, but *ET Geowizards*, an add-on software for ArcGIS, is also used for automatically recording the edges of the river network in the attribute table. Were *ET Geowizards* not used, edge recording would have to be done manually. [↑](#footnote-ref-2)
